# Bacillus Calmette-Guérin (BCG) Vaccine in America and Overseas: A Narrative Review

**DOI:** 10.7759/cureus.73602

**Published:** 2024-11-13

**Authors:** Alan D Kaye, Trevor P Giles, Emily O'Brien, Jennifer Zajac, Willam C Upshaw, Kyle Jenks, Prakriti Arya, Adam M Kaye, Shahab Ahmadzadeh, Debbie Chandler, Sahar Shekoohi, Giustino Varrassi

**Affiliations:** 1 Department of Anesthesiology, Louisiana State University Health Sciences Center, Shreveport, USA; 2 School of Medicine, St. George's University, West Indies, GRD; 3 School of Medicine, Louisiana State University Health Sciences Center, Shreveport, USA; 4 School of Medicine, St. George’s University, West Indies, GRD; 5 Department of Internal Medicine, Doctors Hospital at Renaissance, McAllen, USA; 6 Department of Pharmacy Practice, Thomas J. Long School of Pharmacy and Health Sciences, University of the Pacific, Stockton, USA; 7 Department of Pain Medicine, Fondazione Paolo Procacci, Rome, ITA

**Keywords:** bacillus calmette-guerin, bcg, tb, tuberculin skin test, tuberculosis, vaccine

## Abstract

This narrative review examines the role of the Bacillus Calmette-Guérin (BCG) vaccine in global tuberculosis (TB) control efforts, with particular emphasis on the differences in vaccination policies between countries, such as the US, where routine BCG administration is not practiced. A significant complication of the BCG vaccine is false positive results in the tuberculin skin test (TST), often leading to misdiagnoses and unnecessary treatments. To address these issues, interferon-gamma release assays (IGRAs) have emerged as a more specific diagnostic tool that reduces false positives associated with prior BCG vaccination. However, despite advancements, public health challenges persist in accurately detecting latent and active TB, particularly in populations previously vaccinated with BCG. This review synthesizes existing literature to assess these challenges, emphasizing the need for updated policies and improved diagnostic tools for BCG-vaccinated populations in the US and globally. Recommendations include integrating IGRAs into routine practice and tailoring TB control strategies to mitigate the diagnostic complications of BCG.

## Introduction and background

Tuberculosis (TB) remains one of the world's deadliest infectious diseases despite advances in diagnosis and treatment. The World Health Organization reports that TB caused more than 10 million new infections and 1.5 million deaths in 2021 alone, making it one of the leading causes of mortality worldwide [1,2]. TB primarily spreads through airborne transmission, and although treatment with antibiotics can cure the disease, early detection is essential for adequate control. As the global epidemiology of TB becomes more complex, the need for reliable and accessible diagnostic tools has become increasingly apparent [3].

The Bacillus Calmette-Guérin (BCG) vaccine, developed over a century ago, remains a vital tool in TB prevention. It is particularly effective in reducing TB-related morbidity in children by preventing severe forms of the disease, such as TB meningitis and miliary TB. However, the vaccine's efficacy in preventing adult pulmonary TB varies significantly across regions [4]. The BCG vaccine is routinely administered to newborns in countries with high TB incidence. In contrast, low-burden countries, like the United States, have discontinued routine BCG vaccination mainly due to its limited efficacy in adults and the risk of interfering with diagnostic tests, notably the tuberculin skin test (TST) [5].

The disproportionate global burden of TB is concentrated in low- and middle-income countries, where social determinants, such as overcrowding, malnutrition, and poor access to healthcare, exacerbate the spread of the disease [6]. TB control in these regions is further complicated by the limitations of traditional diagnostic tools, particularly the TST, which frequently yields false-positive results in individuals who have received the BCG vaccine. Newer diagnostic tools, such as interferon-gamma release assays (IGRAs), offer greater specificity by targeting *Mycobacterium tuberculosis*-specific antigens not present in the BCG vaccine. However, these tests are costly and less accessible in low-resource settings, limiting their broader application. The historical development of the BCG vaccine dates back to 1921 when French scientists Albert Calmette and Camille Guérin successfully cultured a strain of *Mycobacterium bovis* that had lost its virulence but retained its capacity to stimulate an immune response [7]. Since then, the BCG vaccine has become one of the most widely used vaccines globally, with over 100 million children receiving it yearly [5]. Its application, however, varies depending on the region, with high-burden countries administering the vaccine universally, while countries with low TB incidence, like the United States, use the vaccine more selectively [8].

Therefore, the present investigation aims to comprehensively examine TB as a global health issue, focusing on the BCG vaccine's historical development, its role in TB prevention, and its diagnostic challenges. By synthesizing existing literature, this review will highlight current diagnostic obstacles and propose recommendations to improve TB control efforts in BCG-vaccinated populations worldwide.

## Review

The role of the BCG vaccine in TB prevention

BCG Vaccine and Its Mechanism of Action

The Bacillus Calmette-Guérin (BCG) vaccine, developed in 1921, remains the only vaccine for tuberculosis (TB) prevention. While its efficacy varies widely, the BCG vaccine has proven especially effective in protecting against severe forms of TB, such as TB meningitis and disseminated (miliary) TB in children [7]. The protective efficacy of the BCG vaccine in adults, particularly against pulmonary TB, is more variable, with studies reporting protective rates ranging from 0% to as high as 80%. This variability is influenced by geographic location and strain variations [9].

The BCG vaccine is derived from a live attenuated strain of *Mycobacterium bovis*, which causes cattle TB and can infect humans [10]. The attenuation process weakens the bacterium's virulence, enabling it to elicit an immune response without causing active disease. The vaccine induces cell-mediated and humoral immune responses, with T-cell immunity playing a crucial role in containing *M. tuberculosis* infections [11].

Differences between BCG strains used in various countries

Globally, different strains of the BCG vaccine exist, each named after its development location, such as BCG Tokyo and BCG Denmark. These strains exhibit distinct genetic and phenotypic characteristics due to variations in the attenuation process, leading to incompletely understood differences in vaccine-induced immune responses [12]. For instance, BCG Pasteur strains contain between 37,500 and 500,000 culturable particles per dose, while BCG Copenhagen strains contain 200,000 and 3.2 million particles. Such differences in vaccine composition may lead to varying immune responses among populations, contributing to differences in vaccine efficacy across regions [13].

Environmental factors, including exposure to non-tuberculous mycobacteria, may influence BCG's efficacy. Individuals residing in areas where environmental mycobacteria are prevalent may possess pre-existing immunity that interacts with the BCG vaccine, potentially diminishing its protective effects [3]. Consequently, the BCG vaccine's protective efficacy varies widely based on geographic location, population exposure, and the specific vaccine strain utilized [12].

Global BCG Vaccination Policies and Practices

Global policies regarding BCG vaccination differ markedly depending on TB incidence and public health priorities. Of the 180 countries with available data, 157 have universal BCG vaccination programs for infants to prevent severe forms of TB [14]. Some nations, such as the United Kingdom, recommend a single BCG dose at birth, while others, like Brazil, endorse multiple doses during early childhood. Conversely, 23 countries, including the United States and several Western European nations, either never adopted universal BCG vaccination or discontinued it due to decreasing TB incidence [15].

In high-TB-incidence countries, such as India and South Africa, universal BCG vaccination is regarded as a vital public health strategy to reduce TB-related mortality in children [16]. Conversely, low-incidence countries like the United States have chosen not to include BCG vaccination in their routine immunization schedules, opting instead to focus on targeted TB screening and treatment for high-risk populations, such as healthcare workers and recent immigrants from high-burden regions [15].

Impact of National Immunization Programs on TB Incidence Rates

The effectiveness of national immunization programs can be assessed through their impact on TB incidence rates. Countries with robust BCG vaccination policies, such as South Africa and India, have documented significant declines in severe childhood TB cases, illustrating the vaccine's role in reducing morbidity and mortality associated with TB [17]. In contrast, countries that have reduced or discontinued BCG vaccination often report challenges in managing TB outbreaks, underscoring the importance of maintaining vaccination coverage in high-risk populations [18].

BCG Coverage in the United States

In the United States, BCG coverage has historically been limited, reflecting the country's low TB prevalence. Per the Centers for Disease Control and Prevention (CDC), routine BCG vaccination is not recommended for the general population due to the low incidence of TB and the potential for misleading results on TB tests [15]. The CDC advises that individuals at high risk for TB exposure, such as healthcare workers or those living in congregate settings, may be considered for BCG vaccination on a case-by-case basis [18]. This selective approach highlights the challenges in balancing TB prevention with the realities of low incidence rates and the complexities of immunological responses [12].

Additional Benefits of BCG Vaccination

Recent studies indicate that the BCG vaccine may confer protective effects beyond TB prevention. Evidence suggests that the BCG vaccine might offer nonspecific protection against other infections, particularly in neonates and the elderly, by enhancing innate immune responses [15]. Additionally, the BCG vaccine is well-established in preventing and treating non-muscle invasive bladder cancer (NMIBC) [1]. It is administered intravesically and functions as an immunotherapy by stimulating the immune system to attack bladder cancer cells, reducing recurrence rates and delaying disease progression [10]. The exact mechanisms through which BCG exerts its antitumor effects involve a combination of direct cytotoxicity and the activation of innate and adaptive immune responses, including the recruitment of immune cells such as macrophages, neutrophils, and T lymphocytes to the bladder wall [5]. Clinical trials have demonstrated that BCG immunotherapy is particularly effective in patients with high-risk NMIBC, significantly lowering the risk of tumor recurrence compared to other therapies. These findings underscore the broader immunological benefits of BCG vaccination, even in countries with low TB incidence [5].

Complications of TB Detection in BCG-Vaccinated Populations

A primary complication stemming from widespread BCG vaccination is its effect on TB detection, particularly in populations where the vaccine is routinely administered. The TST, known as the Mantoux test, remains one of the oldest and most widely used diagnostic tools for TB infection [19]. However, individuals vaccinated with BCG frequently present with false-positive results when undergoing the TST, complicating test interpretation and resulting in unnecessary follow-up investigations [20]. In this regard, a meta-analysis of studies conducted in BCG-vaccinated populations revealed that TST sensitivity and specificity are significantly influenced by prior vaccination, with false-positive rates ranging from 30% to 90% [21]. This issue is particularly pronounced in regions where the BCG vaccine is routinely administered, leading to misdiagnoses of active TB and subsequent public health challenges [3].

TB detection techniques

*Tuberculin Skin Test (TST* *)*

The TST involves an intradermal injection of purified protein derivative (PPD) tuberculin, typically into the forearm. The test site is examined 48 to 72 hours later for any induration. The results are interpreted per the size of the induration: >15 mm is considered positive for the general population, >10 mm is positive for high-risk groups (e.g., healthcare workers), and >5 mm is positive for immunocompromised individuals [19]. The immune response elicited by the vaccine in BCG-vaccinated individuals can complicate the interpretation of TST results, leading to false positives. This limitation highlights the challenges of relying solely on TST for TB detection in populations with high BCG vaccination rates [22].

Interferon-Gamma Release Assays (IGRAs)

In response to the limitations of the TST, newer diagnostic tools, such as IGRAs, have been developed. IGRAs are blood tests that measure the immune response to specific TB antigens by assessing interferon-gamma production. Unlike the TST, IGRA results are not affected by prior BCG vaccination, making them a more reliable option for detecting TB infection in vaccinated individuals [3]. However, it is essential to note that while IGRAs can indicate prior exposure to TB, they do not differentiate between latent TB infection and active TB. This aspect emphasizes the need for comprehensive diagnostic approaches to manage TB cases effectively [5].

Impact of BCG vaccination on TB detection

*False-Positive Results From TST* *in BCG-Vaccinated Individuals*

The administration of the BCG vaccine complicates traditional TB detection methods, particularly the TST. Individuals who have been vaccinated with BCG often present with false-positive results when subjected to the TST. This complication makes it difficult to distinguish between actual infection and prior vaccination, leading to potential overdiagnosis of active TB [21]. The immune response induced by the BCG vaccine can misinterpret TST results, complicating diagnosis and potentially leading to unnecessary treatment or follow-up investigations [23].

*Challenges in Differentiating Latent TB* *Infection From Prior Vaccination*

The immune response to latent TB infection may be indistinguishable from that induced by the BCG vaccine in individuals, mainly when symptoms are absent. As a result, differentiating between latent TB infection and an immune response due to BCG vaccination becomes challenging. Although IGRAs offer a more reliable alternative for vaccinated individuals, they cannot distinguish between active and latent TB infection [3]. Understanding these dynamics is crucial for effective TB diagnosis and public health strategies, especially in populations where BCG vaccination is standard [23].

Comparisons of TST and IGRAs in BCG-vaccinated vs. non-vaccinated populations

Sensitivity and Specificity of Each Diagnostic Method

When comparing the TST and IGRA in BCG-vaccinated versus non-vaccinated populations, several vital factors include accuracy, interpretation, and utility in different contexts. TST sensitivity and specificity are significantly influenced by prior vaccination, which affects its performance in BCG-vaccinated individuals. In contrast, IGRAs maintain higher sensitivity and specificity regardless of vaccination status. A random-effects model meta-analysis has shown that IGRAs are significantly more sensitive than the TST [3].

Effectiveness of IGRAs in Overcoming BCG Interference

IGRAs utilize antigens specific to *Mycobacterium tuberculosis*, which are absent in the BCG vaccine. Therefore, IGRAs can accurately differentiate between an actual TB infection and an immune response induced by BCG vaccination. Research has shown that IGRAs maintain high sensitivity and specificity for detecting latent TB infection (LTBI), even in populations with high rates of BCG vaccination [23]. Numerous studies have demonstrated the effectiveness of IGRAs in overcoming BCG interference, making them a valuable tool in TB detection and management. Both TST and IGRAs have their advantages and disadvantages. The selection of tests often depends on the clinical context, patient history, and availability of resources. In some cases, both tests may be used for a more comprehensive assessment of TB infection.

Implications for public health challenges in TB detection in America

*Low Prevalence of TB in the US*​​​​​​​* and Its Effect on Diagnostic Strategies*

TB, once a significant public health threat, has significantly declined in the United States. This reduction in incidence is primarily attributed to advancements in social conditions, medical care, and the availability of effective antibiotic treatments. The US CDC reported an incidence rate of 2.9 cases per 100,000 individuals in 2023, starkly contrasting the high numbers in the 19th and early 20th centuries [24]. Despite the reduced prevalence, TB remains a critical public health concern due to the potential for LTBI to reactivate, especially among immunocompromised individuals.

Related to this low prevalence, the diagnostic strategies for TB in the US are carefully tailored. The primary screening method remains the TST, which involves injecting tuberculin into the forearm and observing the immune response. This test is beneficial for detecting latent infections, which may otherwise go unnoticed due to the absence of symptoms. However, the TST has limitations, particularly regarding individuals vaccinated with the BCG vaccine, which can lead to false-positive results [24]. These false positives can complicate efforts to accurately identify infected individuals, potentially leading to unnecessary follow-up investigations and treatment.

Addressing the Issue of BCG Interference in Immigrant Populations

One of the most significant challenges for TB detection in the US arises from immigrant populations, particularly those hailing from regions where the BCG vaccine is routinely administered. The BCG vaccine is used globally to prevent severe forms of TB, particularly in children. However, its administration can complicate the results of the TST. Immigrants from countries with high TB prevalence, such as India, South Africa, and certain Southeast Asian nations, often present positive TST results, even without active or latent TB infection. This is because the immune response triggered by the BCG vaccine can mimic the response seen in actual TB infection, making it challenging to interpret TST results accurately [22].

To address this issue, the IGRA has emerged as a more specific alternative to the TST. IGRAs measure the immune system's response to TB-specific antigens that are not found in the BCG vaccine, significantly reducing the likelihood of false-positive results in vaccinated individuals. This test is increasingly used for screening immigrants from high TB burden regions, as it provides a more reliable method for detecting actual TB infections. In cases where either the TST or IGRA yields positive results, further diagnostic steps are necessary to determine whether the individual has active or latent TB [24].

TB Screening Complications for Travelers and Immigrants From BCG-Vaccinated Countries

Globally, TB remains a significant public health threat, particularly in regions with high incidence rates, such as Southeast Asia, Sub-Saharan Africa, and parts of the Western Pacific. In these areas, the BCG vaccine is administered routinely as part of national immunization programs to reduce the morbidity and mortality associated with TB in children [8]. While the vaccine has proven effective in preventing severe forms of TB, such as TB meningitis, its use complicates TB detection, particularly when individuals from these regions travel or immigrate to low-incidence countries like the US. The use of the TST is problematic for travelers and immigrants arriving in the US from BCG-vaccinated countries due to the likelihood of false-positive results. This can lead to unnecessary medical interventions and strain public health resources. Additionally, differentiating between latent TB infection and prior BCG vaccination is difficult, particularly in asymptomatic individuals. This challenge has prompted public health authorities to prioritize the use of IGRAs, which, as mentioned, are unaffected by prior BCG vaccination [6].

*Global Strategies for Accurate TB*​​​​​​​* Detection Amidst Varying Vaccination Policies*

Given the widespread use of the BCG vaccine in high-incidence countries and its difficulties in TB detection, global health authorities have adopted various strategies to improve diagnostic accuracy. For instance, the World Health Organization (WHO) recommends that in countries with low TB incidence, IGRAs are preferable for screening individuals from BCG-vaccinated populations. This approach helps avoid the complications associated with interpreting TST results and allows for more targeted TB prevention efforts [20]. In addition to diagnostic improvements, many countries have implemented robust TB control programs, including vaccination and active surveillance, contact tracing, and treatment for active and latent TB cases. Combining these strategies has helped reduce TB incidence in many high-burden countries. However, the global burden of TB remains substantial, with an estimated 10.6 million people affected by the disease in 2021 alone [5].

In conclusion, while the BCG vaccine plays a vital role in protecting against severe TB in high-incidence countries, its interference with traditional TB diagnostic methods presents a challenge, particularly in low-incidence countries like the US, where the vaccine is not routinely administered. The advent of IGRAs has provided a valuable tool for overcoming these challenges, enabling more accurate detection of TB infections in populations with a history of BCG vaccination. Moving forward, continued improvements in diagnostic techniques and comprehensive TB control programs will be critical for managing and ultimately reducing the global burden of TB.

## Conclusions

The findings of this narrative review underscore the critical role of the BCG vaccine in global TB prevention while highlighting its significant interference in TB detection. Specifically, the BCG vaccine complicates using the TST, often leading to false-positive results in vaccinated individuals. This challenge is particularly pronounced in regions where universal BCG vaccination is implemented, leading to misdiagnoses and unnecessary treatment of individuals who may not be infected with *Mycobacterium tuberculosis*. Consequently, the need for more accurate diagnostic tools has become paramount in populations where BCG vaccination is prevalent. The introduction of IGRAs marks a significant advancement in TB detection for BCG-vaccinated populations. IGRAs, such as QuantiFERON-TB Gold and T-SPOT.TB, offer a higher specificity by targeting *M. tuberculosis*-specific antigens absent in the BCG vaccine. As a result, IGRAs minimize the risk of false-positive results that are often associated with TST, providing a more reliable method for distinguishing between individuals infected with TB and those who are not. This development has proven particularly valuable in high-burden countries where the BCG vaccine is routinely administered and in low-burden countries like the US, where TST is widely used in TB screening programs. In addition to its role in TB prevention, the BCG vaccine has shown remarkable effectiveness in preventing and treating bladder cancer. As an immunotherapeutic agent, BCG is widely used in treating non-muscle invasive bladder cancer, significantly decreasing the risk of recurrence and progression, making it an essential tool in urologic oncology. These findings further emphasize the versatile applications of the BCG vaccine, not only in infectious disease control but also in cancer prevention.
